# A Comparison of Functional Outcomes and Cost of Rehabilitation Treatment in the Conservative Treatment of Distal Radius Fractures in a Geriatric Population Between Two Different Wrist Joint Immobilization Positions at One‐Year Follow‐Up

**DOI:** 10.1155/aort/9949821

**Published:** 2025-12-17

**Authors:** Grigorios Kastanis, Mikela- Rafaela Siligardou, Constantinos Chaniotakis, Alexandros Tsioupros, Ioannis Stavrakakis, Petros Kapsetakis, Ioannis Ktistakis, Anna Pantouvaki

**Affiliations:** ^1^ Department of Orthopedic, Venizeleio General Hospital, Heraklion, Crete, Greece, venizeleio.gr; ^2^ Department of Physiotherapy, Venizeleio General Hospital, Heraklion, Crete, Greece, venizeleio.gr

**Keywords:** Colles’fractures, conservative treatment, geriatric distal radius fractures, pain, rehabilitation, treatment outcomes

## Abstract

**Introduction:**

Distal radius fracture (DRF) is the most common fall‐related fracture, with an incidence of up to 18% of the elderly population who are being examined in the emergency department. Conservative treatment in the geriatric population is the treatment of choice, and traditionally the wrist after reduction is placed in volar flexion and ulnar deviation position (Cotton position). The aim of this study is to compare two‐cast position (Cotton and Functional position) for conservative treatment of geriatric DRFs, according to functional outcomes at twelve months.

**Patients and Methods:**

This randomized prospective study compared and evaluated the functional outcomes and cost of physiotherapy in the geriatric population with DRFs. Regarding functional outcomes, these were measured using the QuickDASH Score, Patient‐Reported Wrist Evaluation, pain (VAS score), and health‐related quality of life measurement (15D), while for the cost of physical therapy, the number of sessions performed by patients in both groups was measured.

**Results:**

Ninety‐three patients (75 female and 18 men) with an average age 79.2 ± 6 (range 67–90 years) with a DRF were included in the study. Among these patients, 9 underwent surgical intervention due to loss of reduction and were consequently excluded from our study. The study ultimately encompassed a total of 84 patients. The mean age for Group A was 79 ± 2 years, and for Group B, it was 79 ± 1 years (*p* = 0.61). The mean follow up for all patients was 1 year. Functional cast‐position group (Group B) showed better results in terms of functional recovery: PRWE (Mdiff = 1.52, 95% CI [−7.77, 3.81]), QuickDASH Score (Mdiff = 8.00, 95% CI [2.27, 13.72]), and posttraumatic pain (Mdiff = 1.27, 95% CI [0.86, 1.69]). Cost of physiotherapy (*z* = 128, *p* < 0.001) and HRQol‐15(Mdiff = 1.81, 95% CI [1.02, 2.60]) was statistically significantly greater in the Cotton position group versus Functional position group.

**Conclusion:**

Our results indicate that functional cast‐position produces better functional outcomes with a lower rate of complication than volar‐flexion and ulnar‐deviation cast (VFUDC) position. Despite the fact that the VFUDC group underwent a greater number of physical therapies, they presented less good functional results. In conclusion we recommend the use of functional cast in elderly low energy DRFs.

## 1. Introduction

Distal radius fractures (DRFs) are the most common fall‐related fracture, with an incidence of 18% in the geriatric population treated in the emergency department [1]. Risk factors for DRFs in the elderly include osteoporosis, low body mass index, tendency to fall, and weather conditions (e.g., snow) [2, 3]. Oyen et al. suggest that patients with low‐energy DRFs are at increased risk for subsequent hip and spinal fractures [3]. The typical cause of DRF in the elderly is a fall from a standing height onto an outstretched hand. Regarding fracture type, the majority are dorsally angulated fractures (Colles’ fractures) [4].

Conservative treatment with cast immobilization remains the treatment of choice for stable DRFs in the geriatric population [5]. The main objective of the cast is to maintain fracture alignment and stability until healing occurs. In the elderly, decreased bone mineral density may lead to instability of the fracture, resulting in secondary displacement, which has been reported in 30%–50% of cases after closed reduction. The severity of redisplacement is correlated with increasing age [6].

Many methods of immobilization for DRFs have been proposed in the literature, with varying functional outcomes, but the most appropriate method remains unclear. Cotton GF first introduced stabilization of the DRF in a volar‐flexion and ulnar‐deviation cast (VFUDC) position, using the principle of ligamentotaxis to maintain reduction [7]. Gupta reported that wrist dorsiflexion is the optimal functional position to stabilize DRFs in elderly patients and avoid stiffness complications associated with prolonged use of Cotton’s position [8]. In a comparative study, Ax et al. evaluated VFUDC versus functional cast (FC) and found that the FC method reduced the risk of hand stiffness during immobilization, while VFUDC was associated with higher treatment costs in patients aged 65 and older [4].

The aim of this study is to compare the VFUDC and FC methods for the conservative treatment of geriatric DRFs (Colles’ fractures), with respect to functional outcomes and the cost of the rehabilitation program at twelve months.

## 2. Materials and Methods

This study was performed at the Orthopedic department of Venizeleio General Hospital of Heraklion from January 2022 to February 2023. All patients were fully informed about the study’s rationale and design and provided written consent for participation. Patient rights and privacy were strictly safeguarded throughout the study. Additionally, the study protocol underwent thorough evaluation and received approval from the Institutional Review Board of the Venizeleio General Hospital of Heraklion(Approval Number: 9602). All study procedures adhered to the Ethical Principles for Medical Research Involving Human Subjects, as outlined in the Declaration of Helsinki (1964) and its subsequent amendments, including the 2013 revision.

**Figure 1:**
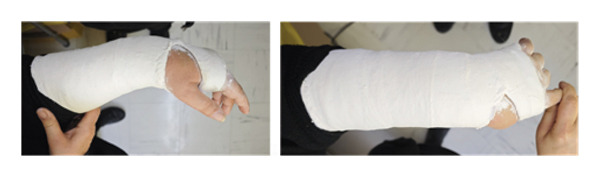
Distal radius fracture immobilized in the volar‐flexion and ulnar deviation position.

**Figure 2:**
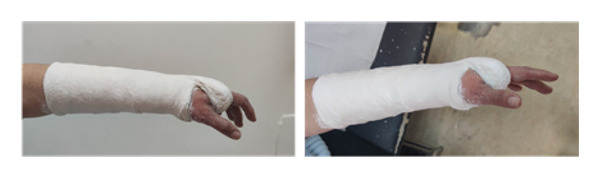
Distal radius fracture immobilized in the functional cast position.

Inclusion criteria were as follows: age > 65 years, low‐energy DRFs, closed extra‐articular or closed partial intra‐articular fractures (A2, B2, and B3 according to the AO classification), and follow‐up interval at least twelve months. Exclusion criteria were as follows: age < 65 years, open fractures, fractures on the initial diagnosis were included in the indications for direct surgical treatment, polytrauma patients, fractures associated with vascular injuries, and pathological fractures or previous fracture in the same wrist. All fractures after radiographic examination (profile and anteroposterior views) were classified by the AO classification system as A2 64 (68.8%) cases, B2 25 (26.9%) cases, and B3 4 (4.3%) cases. After registration of patient characteristics, local anesthesia and closed reduction of the fracture and stabilization randomly in volar‐flexion and ulnar deviation position (group A) and functional position (group B) were performed. Group A consists of 45 patients, in whom the wrist was immobilized in the VFUDC position (Figure 1), while Group B consists of 48 patients, in whom the wrist was immobilized in the FC position (Figure 2). All casts were circumferential, below‐elbow plaster casts. New X‐rays following reduction were performed in order to control the appropriate reduction (shortening, dorsal angulation, and radial inclination were measured) of the fracture. The reductions were performed by a single orthopedic surgeon (GK) from the emergency department to minimize potential variations in individual surgical skills. The other orthopedic surgeons assisted in the reductions under the guidance of the primary surgeon. The mean follow‐up was 1 year, during which the results were assessed based on functional outcomes (QuickDASH Score [QUICΚ DS] and Patient‐Reported Wrist Evaluation [PRWE]), pain (VAS score), and health‐related quality of life.

### 2.1. Follow‐Up

The treatment period with cast was 5 weeks, and radiographic follow‐up was at 1, 2, and 5 weeks. Cast changed in situation in which the patient has symptoms related to cast (numbness of median nerve or swelling of the hand) or the situation in which we are checking loss of fracture reduction. In eleven cases, we observed loss of reduction in a mean period of 7–14 days (6 in Group A and 3 in Group B), and the patients underwent open reduction and volar‐locking plate osteosynthesis according to the indication for surgical treatment (radial shortening > 3 mm, dorsal tilt > 10°, or intra‐articular displacement or step‐off > 2 mm) [9]. The final cohort of patients included in our study comprised 84 individuals. All patients were followed by the same surgeon (GK) who performed the initial reduction in the outpatient clinic. The criteria that ultimately led to surgical intervention are described above. Parameters that fell within these criteria were managed conservatively, in accordance with the aim of the present study. An attempt at reduction was made exclusively. The reduction procedure was performed according to the method described by Charnley J [10]. In seven cases (6 in Group A and one in Group B), the cast had to open but not removed for a week because of the swelling of the hand. Finally, in 5 weeks, we performed removal of the cast and control of fracture consolidation with X‐ray examination.

### 2.2. Rehabilitation

The purpose of DRF rehabilitation is to decrease the functional limitations caused by the fracture or from immobilization in the cast (stiffness). Rehabilitation protocols were performed by the hand therapist, who undertook initial supervised mobilization in cases when signs of excessive edema, disproportional pain, stiffness, sympathetic nervous system activity, or the inability to use the extremity, followed by patients’ education for a daily exercise program [11]. Initially, passive and active movements of the wrist were performed to restore the range of motion, followed by gradual strengthening exercises. In the national health system in Greece, the maximum number of physical therapy sessions financially covered by the state insurance is 20 sessions per year regardless of the condition for which they are performed. If the patient requires a higher number of sessions, they will have to cover the cost themselves. The cost of each session is estimated at 15 euros in state physiotherapy centers. In private centers, the cost is different, but we calculated the public health system’s price. All the patients follow rehabilitation programs in state physical therapy centers or in private ones. The number of physiotherapy sessions was set at 20 for all patients in order to obtain comparable results. Coordination took place between the orthopedic surgeon, the physiotherapist, and the patient to determine whether additional physiotherapy sessions were necessary to achieve the maximum possible outcome.

We evaluated the costs between the two treatment groups by recording the number of sessions of physiotherapists that each patient required to be able to return to their previous functional daily activity.

### 2.3. Statistical Analysis

We performed the statistical analysis using SPSS Version 27.0 (SPSS Inc., Chicago, IL, USA). Continuous variables were expressed as mean ± standard deviation, and categorical variables were represented by frequency and percentage. To test the normality condition, the Shapiro–Wilk test was used in parallel with the study of the “Normal Q‐Q plot,” “Detrended Normal Q‐Q plot,” and “Box Plot” graphs.

Οne‐way ANCOVA was conducted to examine differences in functional outcomes before and after twelve months from the intervention for conservative treatment of geriatric DRFs, between two cast positions (Group A = Cotton position andG group B = Functional position) [12, 13]. We used the Mann–Whitney *U* test to compare differences of physiotherapy cost, between Groups A and B.

For all tests, statistical differences were determined to be significant at *p* < 0.05. In addition to the *p* value, the effect size was used to evaluate the significance of an effect. Effect size is an objective and standardized measurement of the magnitude of the observed effect (the practical significance of the effect). The effect size was calculated based on Cohen’s criteria. In the case of Mann–Whitney *U* test, the effect is low if its value is less than 0.5, medium if it ranges between 0.5 and 0.8, and large if it is greater than 0.8. In the case of one‐way ANCOVA, the effect is low if its value is less than 0.01, medium if it ranges between 0.01 and 0.14, and large if it is greater than 0.14.

## 3. Results

The study sample consisted of 65 women and 19 men, with average age 79.2 ± 6 years. Fracture sides were in the right hand in 60 cases (71.4%) and in the left in 24 cases (28.6%), while the dominant hand was right in 73 (86.9%) cases and left in 11 (13.1%) patients. The mean age for Group A was 79 ± 2 years, and for Group B, it was 79 ± 1 years (*p* = 0.61). Rehabilitation costs were evaluated based on the number of physical therapy sessions the patients required. The mean follow‐up for all patients was 1 year.

### 3.1. QUICΚ DS

**Figure 3:**
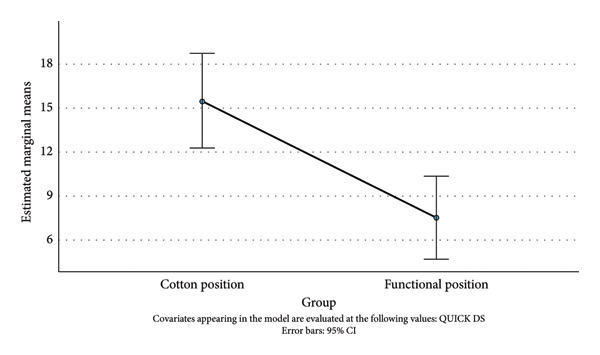
Estimated marginal means of QuickDASH (12 months).

A one‐way ANCOVA was run to determine the effect of a Cotton and Functional position on postintervention QUICK DS after controlling for preintervention QUICK DS. After adjustment for preintervention QUICK DS (22.71), there was a statistically significant difference in postintervention QUICK DS between the cast position groups, *F*(1, 81) = 7.72, *p* = 0.007, and partial *η* ^2^ = 0.087 (medium effect size). Post hoc analysis was performed with a Bonferroni adjustment. Postintervention QUICK DS was statistically significantly greater in the Cotton position group versus Functional position group (Mdiff = 8.00, 95% CI [2.27, 13.72]). That is, in the Functional position group, the decrease in QUICK DS was, statistically significantly, greater than that in the Cotton position group (Figure 3).

### 3.2. PRWE

**Figure 4:**
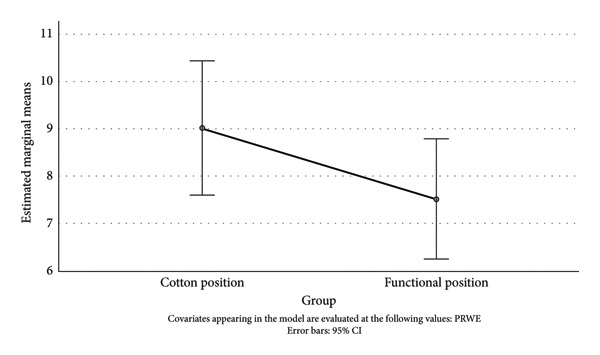
Estimated marginal means of PRWE score (12 months).

A one‐way ANCOVA was run to determine the effect of a Cotton and Functional position on the postintervention PRWE score after controlling the preintervention PRWE score (Figure 4). After adjustment for the preintervention PRWE score (22.56), there was no statistically significant difference in the postintervention PRWE score between the cast position groups, *F*(1, 81) = 1.74, *p* = 0.191. Post hoc analysis was performed with a Bonferroni adjustment. Postintervention PRWE score was not statistically significantly different in the Cotton position group versus Functional position group (Mdiff = 1.52, 95% CI [−7.77, 3.81]).

### 3.3. Pain (VAS Score)

**Figure 5:**
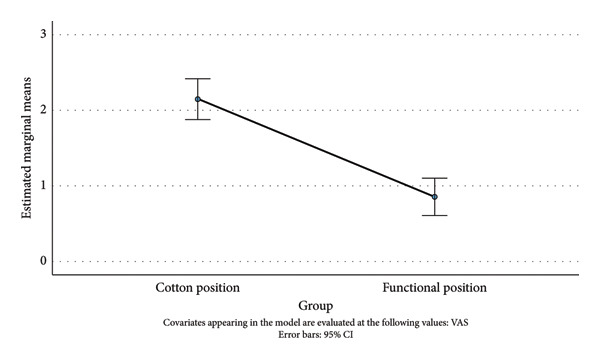
Estimated marginal means of VAS (12 months).

A one‐way ANCOVA was run to determine the effect of a Cotton and Functional position on the postintervention VAS score after controlling for the preintervention VAS score. After adjustment for the preintervention VAS score (3.54), there was a statistically significant difference in the postintervention VAS score between the cast position groups, *F*(1, 81) = 37.12, *p* < 0.001, and partial *η* ^2^ = 0.314 (large effect size). Post hoc analysis was performed with a Bonferroni adjustment. The postintervention VAS score was statistically significantly greater in the Cotton position group than that in the Functional position group (Mdiff = 1.27, 95% CI [0.86, 1.69]). That is, in the Functional position group, the decrease in the VAS score was, statistically significantly, greater than that in the Cotton position group (Figure 5).

### 3.4. Health‐Related Quality of Life (HRQoL‐15)

**Figure 6:**
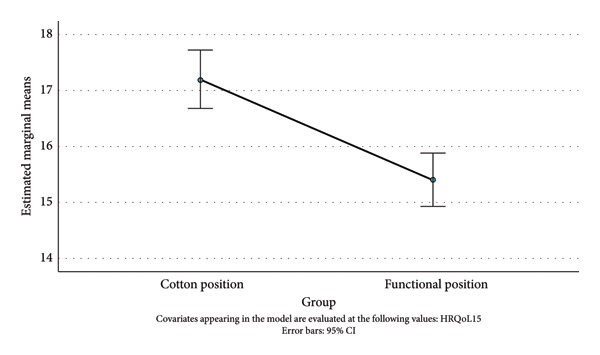
Estimated marginal means of HRQoL‐15 Score (12 months).

A one‐way ANCOVA was run to determine the effect of a Cotton and Functional position on the postintervention HRQoL‐15 score after controlling for the preintervention HRQoL‐15 score. After adjustment for the preintervention HRQoL‐15 score (18.98), there was a statistically significant difference in the postintervention HRQoL‐15 score between the cast position groups, *F*(1, 81) = 20.98, *p* < 0.001, and partial *η* ^2^ = 0.206 (large effect size). Post hoc analysis was performed with a Bonferroni adjustment. The postintervention HRQoL‐15 score was statistically significantly greater in the Cotton position group than that in the Functional position group (Mdiff = 1.81, 95% CI [1.02, 2.60]). That is, in the Functional position group, the decrease in the HRQoL‐15 score was, statistically significantly, greater than that in the Cotton position group (Figure 6).

### 3.5. Physiotherapy Cost (Physical Therapy Sessions)

**Figure 7:**
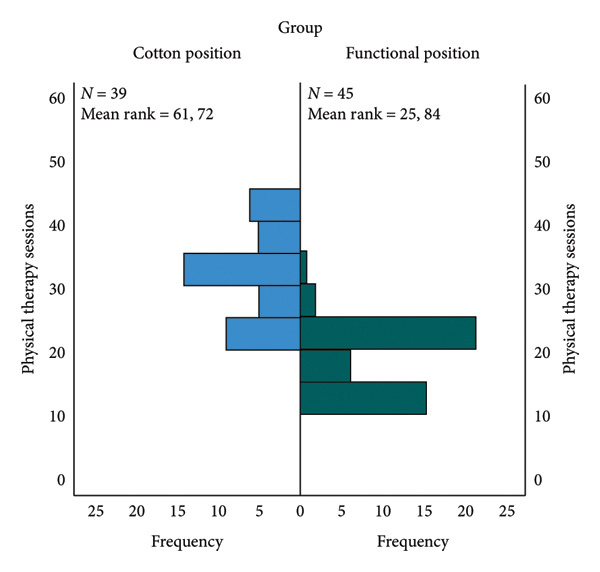
Physical therapy sessions per group.

A Mann–Whitney *U* test was performed to compare physical therapy sessions in the Cotton position group and the Functional position group. There was a significant difference in physical therapy sessions between the two groups: *z* = 128, *p* < 0.001. In the Cotton position group, the cost of physiotherapy is, statistically significantly, higher than that in the Functional position group (Figure 7).

## 4. Discussion

DRF is very common injury in the geriatric population with an incidence rate of between 200 and 1200 per 100,000 person per year [2]. In patients over 65 years, DRF is the second most common fracture after hip fractures, while over the age of 75 years, DRF constitutes the third most common fracture after hip and proximal humerus fracture [11, 14]. The most common mechanism of injury in geriatric DRF is a fall from a standing height over an outstretched hand [14].

It has been mentioned in the literature that the risk factors for DRF vary according to age, gender, and living conditions, and that women have an increased risk of DRF compared with men [11]. Oyen et al. mention that osteoporosis is higher in elderly population and consequently can be consider the main risk factor for both men and women, while Brogren et al. mention that the incidence of DRF fracture among woman increased with age and not with menopause [3, 15]. Moreover, Davies et al. reported that the risk of falling increased after menopause among women, possibly due to poor reaction time and reduced muscle strength [16].

Regarding the type of fracture and displacement, the majority of geriatric DRFs are extraarticular with dorsal angulation (Colles’ fracture) and are generally considered stable fractures [6, 15]. A stable fracture is considered a fracture that, after reduction, retain the alignment or has a low probability of displacement [6]. Secondary displacement factors have also been identified as age over 60, dorsal angulation greater than 20°, 5‐mm radial shortening, dorsal comminution, concomitant ulna fracture, and radiocarpal involvement [6]. Geriatric patients have decreased bone mineral density, which causes DRF instability and presents an incidence of 30%–50% secondary displacement during cast mobilization [15, 17]. In our study, the majority of the fractures were extraarticular AO Type A2 (64‐68.9%), and only 11(11.9%) cases presented with secondary displacement and underwent surgical treatment. From these 11 cases, five cases were classified as AO Type A2 (5.4%), 4 as AO Type B2 (4.3%), and 2 as (2.2%) AO Type B3.

The objective of the treatment is to recapture function to a level as closer to the prefracture level of function as possible. Despite the treatment method, some patients will still experience pain and stiffness to some extent [11]. Traditional closed reduction of the fracture near‐anatomical position followed by immobilization in a plaster cast for 4‐5 weeks, remains the most accepted treatment method for geriatric stable DRF [6, 11].

Regarding the immobilization position of the wrist (with the aim of maintaining the reduction and bone consolidation) for conservative treatment of DRF, several methods have been proportioned. Frederic J. Cotton (1910) first mentioned the VFUDC of the wrist position, while Charnley J (1950) was the greatest supporter of this position [7]. Τhey argued that a position of flexion and ulnar deviation, wound induce soft tissue around fracture area, and the pull produced by radiocarpal ligaments prevent the dislocated forces generated over the fracture line [5, 7]. In clinical practice, two major complications appear: First, this position causes the common extensor tendons to be under constant tension and produce inappropriate finger flexion during cast therapy. This prolonged position leads to stiffness of the joints of the hand and wrist [5, 18]. Second, the pressure in the carpal tunnel increases from 18 to 47 mmHg, leading to median nerve compression [19].

Gupta A (1991) introduced the dorsiflexion position of the wrist to avoid these complications. In his research, he compared three immobilization positions of the wrist in DRF (VFUDC, FC, and dorsiflexion) and concluded that immobilization of the wrist in dorsiflexion provide better maintenance of reduction and decreases the risk of secondary redisplacement of the fracture [8]. Van Delft et al. in a systematic review reported better functional results in patients who were treated with cast immobilization in dorsiflexion, while Baruah et al. added that dorsiflexion of the wrist enhances the rehabilitation of fingers during the treatment [20, 21].

Recently, Ax et al. in a randomized, multicenter study evaluated the functional results and costs of treatment of the two casting positions (VFUDC and FC) in patients aged 65 and above with DRF [4]. They report better functional results in FC group patients, while cost analysis (physical therapy and surgery) between the two groups revealed double cost in the VFUDC group. Regarding physiotherapy, an increased number of sessions were reported compared with FC and had consistently slightly inferior functional results [4]. In our study, we found the same functional outcomes in the FC group than in VFUDC (PRWE (Mdiff = 1.52, 95% CI [−7.77, 3.81]) and QUICK DS (Mdiff = 8.00, 95% CI [2.27, 13.72]) and a lower complication rate in the FC group. FC can provide similar functional recovery as VFUDC, with fewer complications, supporting its consideration as an effective and safe option for this type of fracture. Carpal tunnel syndrome was described in 8 cases in VFUDC and only in 3 cases in FC. No patient reported median nerve compression symptoms before the fracture in VFUDC, while two cases in the FC group reported having a mild grade. Regarding cost of the physiotherapy session, it was almost double in VFUDC compared with FC *z* = 128, *p* < 0.001.

Our study has the following limitation: (1) small sample of patients (however, we believe that it has to provide some data that will not change even with a larger sample of patients). (2) Age difference between the patients: It is possible that if we categorize the patients by decade, we may have different results. (3) Short follow‐up and recording of results. 4. Classification of the DRFs to be the same of a category regarding the classification of AO.

## 5. Conclusion

We found a consistent difference in functional results between the volar‐flexion and ulnar deviation position and FC position groups. Our results postulated that FC produces better functional outcomes in 1 year with a lower rate of complication than VFUDC. According to the cost analysis of the number of physiotherapy sessions, while the VFUDC group underwent a greater number of physical therapies, they presented less good functional results. We recommend the use of FC in elderly patients with stable DRFs.

## Conflicts of Interest

The authors declare no conflicts of interest.

## Author Contributions

Grigorios Kastanis: conceptualization, formal analysis, data curation, investigations, methodology, supervision, validation, visualization, writing–original draft, and writing–review and editing.

Mikela‐ Rafaela Siligardou: conceptualization, formal analysis, data curation, investigations, methodology, validation, visualization, writing–original draft, and writing–review and editing.

Constantinos Chaniotakis: conceptualization, formal analysis, data curation, investigations, methodology, supervision, validation, visualization, writing–original draft, and writing–review and editing.

Alexandros Tsioupros: conceptualization, formal analysis, data curation, investigations, methodology, validation, visualization, writing–original draft, and writing–review and editing.

Ioannis Stavrakakis: conceptualization, formal analysis, data curation, investigations, methodology, validation, visualization, writing–original draft, and writing–review and editing.

Petros Kapsetakis: conceptualization, formal analysis, data curation, investigations, methodology, validation, visualization, writing–original draft, and writing–review and editing.

Ioannis Ktistakis: conceptualization, formal analysis, data curation, investigations, methodology, validation, visualization, writing–original draft, and writing–review and editing.

Anna Pantouvaki: conceptualization, formal analysis, data curation, investigations, methodology, validation, visualization, writing–original draft, and writing–review and editing.

## Funding

The authors have not received any financial support for this research and publication of this manuscript.

## Data Availability

The data that support the findings of this study are available from the corresponding author upon reasonable request.
